# COVID-19 screening center models in South Korea

**DOI:** 10.1057/s41271-020-00258-7

**Published:** 2020-10-21

**Authors:** Ji Eon Kim, Ji Ho Lee, Hocheol Lee, Seok Jun Moon, Eun Woo Nam

**Affiliations:** 1 Department of Health Administration, Yonsei University Graduate School, Wonju, Republic of Korea; 2 Yonsei Global Health Center, Yonsei University, Wonju, Republic of Korea; 3 Department of Health Administration, Yonsei University, Changjo Hall 412, Yonseidae-gil 1, Wonju, 26493 Republic of Korea

**Keywords:** COVID-19, Screening center, Drive-through screening center, Walking-through screening center, South Korea, Testing

## Abstract

In a recent report, the British Broadcasting Company (BBC) introduced South Korea’s measures to manage COVID-19 as role model for the world. Screening centers serve as frontiers for preventing community transmission of infectious diseases. COVID-19 screening centers in Korea operate 24 h a day, always open for individuals with suspected COVID-19 symptoms. South Korea concentrated COVID-19 screening centers around cities with high population density. Advanced screening centers (models C, D, and E) proved more effective and efficient in the prevention of COVID-19 than the traditional screening centers (models A and B). Particularly, screening centers at Incheon Airport in South Korea prevent transmission through imported cases effectively. It will be important elsewhere, as in South Korea, to establish an infectious disease delivery system that can lead to 'Test-Treat-Track' using an adequate model of screening centers.

## Introduction

Since the first report of COVID-19 from Wuhan, Hubei, China, in December 2019, the spreading of this disease has continued. The World Health Organization (WHO), on 11 March 2020, declared COVID-19 a “Global Pandemic”, at the highest level of alert [1]. The first confirmed case of COVID-19 in South Korea was a 35-year-old Chinese woman diagnosed on 20 January 2020.

As of 31 August 2020, the United States (USA) reported the highest number of confirmed cases (6,173,236), followed by Brazil (3,862,311), India (3,619,169), Russia (990,326), and Peru (647,166) [2]. In South Korea, newly confirmed cases peaked at 813 on 29 February 2020, decreased after, then on 31 April 2020, 81 newly confirmed cases were reported. Although COVID-19 spread later in Europe, the USA, and in Latin America than in East Asian counties, including China, South Korea, and Japan, it is spreading rapidly worldwide since. In a recent report, BBC introduced South Korea's response measures to COVID-19 as a model for the world [3]. TIME published an article suggesting that the USA should use the Korean model to respond to COVID-19 [4]. A fast and effective screening system with COVID-19 screening centers in regions all around the country is the basis of this model.

Other countries have devised varied strategies to respond to COVID-19, including screening centers. Taiwan did not conduct an immediate diagnostic test for those suspected of having COVID-19. Instead, Taiwan’s Centers for Disease Control guided self-quarantine and self-care for 2 weeks for patients suspected to be infected [5]. In Japan, the government decided testing was not essential for individuals who showed no symptoms of COVID-19. Japan’s government changed from a ‘Containment’ prevention strategy (quarantine) to the ‘Suppression’ prevention strategy (with testing) from 7 April 2020. As of 8 April 2020, Japan initiated a cluster infection strategy to protect the population, focusing on high-risk groups [6].

Screening centers serve as frontiers for preventing community transmission of infectious diseases. COVID-19 screening centers in Korea operate 24 h a day, always are open for individuals with suspected of having COVID-19 symptoms. This approach minimizes community contact with those suspected of carrying the infection and prevents community-acquired infections. The Korea Centers for Disease Control and Prevention (KCDC) updates the status of COVID-19 screening centers across the country every day and provides relevant information to people using text messages and internet homepages. KCDC also designates public relief hospitals*,* drive-through (DT) screening centers, clinics that offer COVID-19 sample collection, and all other screening centers.

We believe that other countries will help to conclude the worldwide pandemic as soon as possible if learn from and implement the sort of screening system we studied and discuss—modified to suit specific requirements of the region.

This study aims to:

- Examine the location and distribution of general and drive-through COVID-19 screening centers based on population density in South Korea.
- Classify COVID-19 screening centers by type, compare their characteristics, and provide basic information about establishing screening centers.
- Share the strategies South Korea implemented against COVID-19 with other countries suffering from later outbreaks.

## Materials and methods

### Data collection

South Korea placed its COVID-19 screening centers using data confirmed by the KCDC, as of 18:00 on 30 March 2020. The NASA Socioeconomic Data and Applications Center (SEDAC) of the NASA Earth Science Data and Information System (ESDIS) project provided the data South Korea used for analyzing where to place screening centers [7]. We categorized the types of COVID-19 screening centers in South Korea based on previously published literature, press releases by the government, news articles, case reports, and legal provisions. The study covered all 690 South Korean COVID-19 screening centers established as of 18:00 on 30 March 2020.

### Analysis methods

We ascertained the locations of general and drive-through COVID-19 screening centers in South Korea using the Arc GIS Mapping system, and analyzed their distribution based on regional population density. The research team converted addresses of the 690 COVID-19 screening centers in South Korea into Arc GIS coordinates and mapped all for further analysis. We categorized Regional population density in 5 levels. The lowest level is 1 person or less per 1 km^2^, and the highest level is 1000 people, or more, in 1 km^2^. For mapping regional population density, we assigned the lightest color to the lowest density areas, and the darkest color to the areas with highest levels of density. We categorized the types of COVID-19 screening centers in South Korea and compared their characteristics: location, hours of operation, specimen collection (per hour), health care workers on site (HCW), necessary personal protective equipment, and medical equipment.

## Results

### Location and distribution of COVID-19 screening centers in South Korea

South Korea had 611 “General COVID-19 screening centers” and 79 “Drive-Through (DT) COVID-19 screening centers” as of 18:00 on 26 March 2020. Gyeonggi-do had the highest number (*n* = 108), and Sejong had the lowest number (*n* = 2) of general screening centers. Gyeonggi-do also had the highest number of drive-through screening centers (*n* = 13); Jeju had none. Jeollanam-do had the highest number of screening centers per 10,000 at 0.35, Sejong the lowest at 0.06. Population density was the highest in Seoul with 16,034/km^2^ and the lowest in Gangwon-do with 90/km^2^ [8, 9].

**Fig. 1:**
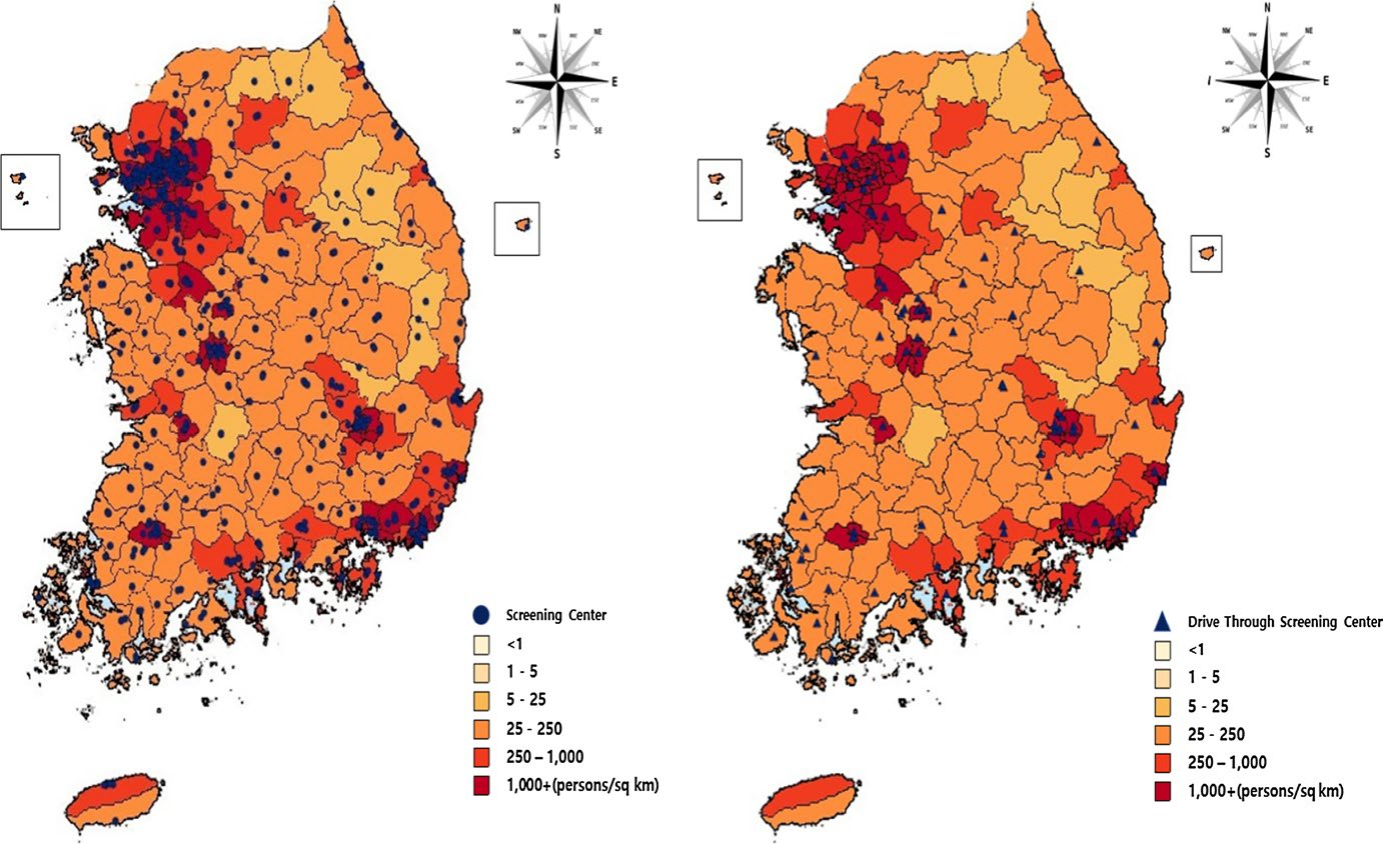
Distribution of general (left) and drive-through (right) COVID-19 screening centers in South Korea

Figure 1 depicts the distribution of general and DT COVID-19 screening centers in South Korea. At least one general COVID-19 screening center operated in each administrative division (Si, Gun, or Gu). Based on the population density in Fig. 1, we found the general COVID-19 screening centers to be most concentrated in Seoul and Gyeonggi-do, where population density was higher than in other regions. South Korea established many COVID-19 screening centers in 6 additional cities, Busan, Incheon, Daegu, Daejeon, Gwangju, and Ulsan.

**Fig. 2:**
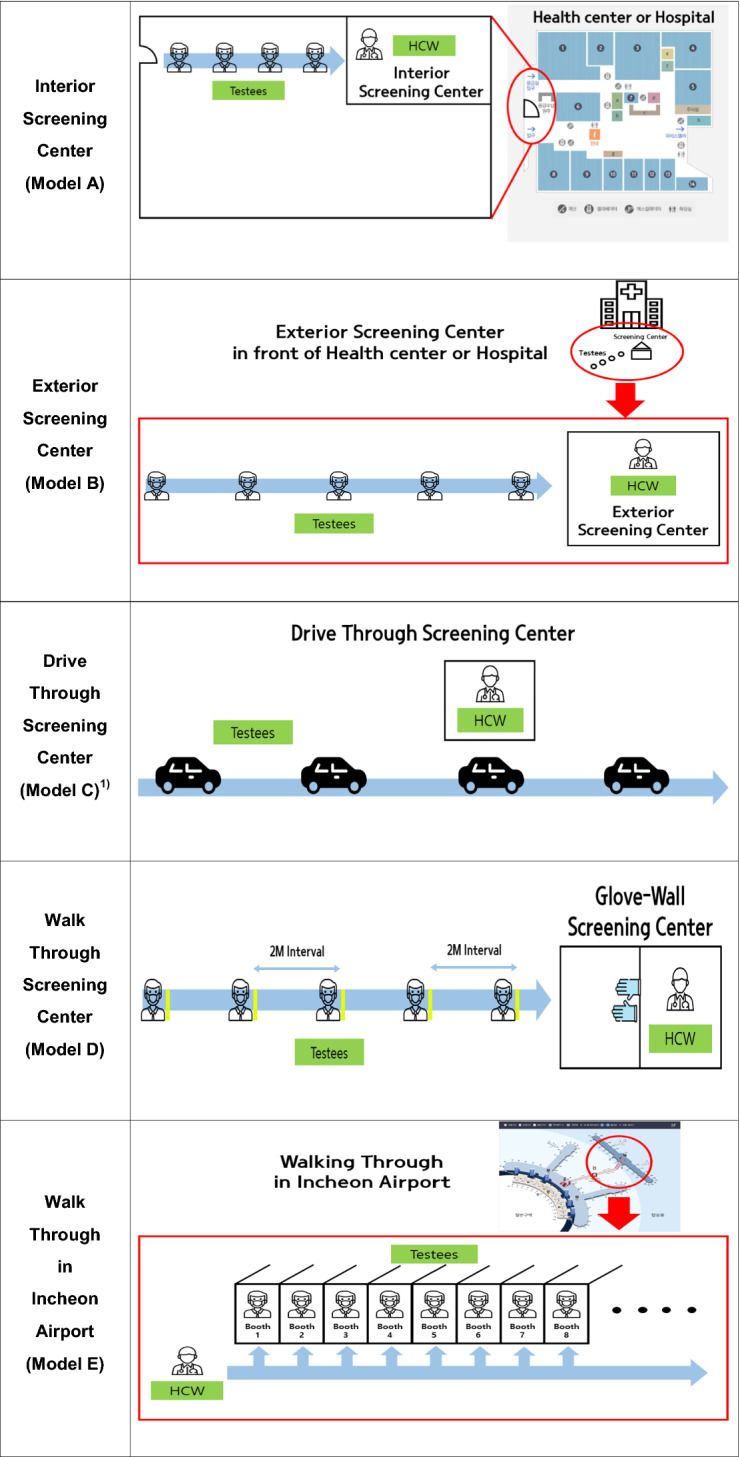
Types of COVID-19 screening centers in South Korea

We found distribution of DT screening centers across Seoul and the surrounding areas of Gyeonggi-do where the population density was high, as it was in the 6 other cities. In the cities with population density of and above 250/km^2^, 22 of 38 did not have DT screening centers. In cities with population density of or above 1000/km^2^, 2 cities of 73 did not have DT screening centers.

### Types and comparison of characteristics of COVID-19 screening centers in South Korea

Figure 2,^1^ presents the types of COVID-19 screening centers present at the time of our study in South Korea. We divided them into the following 5 categories: Interior screening center (Model A), Exterior screening center (Model B), Drive-through screening center (Model C), Walking-through screening center (Model D), and Walk-through in Incheon Airport (Model E).

The characteristics of each model are as follows:

- *Model A* Operates inside hospital buildings and public health centers. Health care workers (HCW) wait inside the screening centers, and those wishing to be tested for COVID-19 line up or use the waiting areas. Model A is the most basic type of screening center, used often to screen for diseases with relatively low risk of transmission.
- *Model B* Operates outside hospital buildings and public health centers. HCWs wait inside the screening centers, and those wishing to be tested must line up outside. This type of center screens for diseases with high risk of transmission, such as those in WHO-defined epidemic situations. COVID-19 screening centers outside hospital buildings and public health centers use negative pressure tents well equipped with portable X-ray machines and COVID-19 testing kits. Negative air pressure prevents leakage of contaminated air from inside the tent to the outside. Because these tents can be used to isolate patients, they help to reduce transmission when HCWs screen suspected patients at early stages for COVID-19 [10].
- *Model C* Operates in parking lots where those wishing to be tested arrive in cars and open their car windows for evaluation of their histories and for monitoring of body temperature [8]. Staff tell those waiting for testing to maintain internal air circulation in their cars, that serve as isolation booths. HCWs visit each car to perform the tests. The cars also serve as negative pressure patient rooms or tents; this also is an effective method to prevent disease transmission [11]. South Korea designed Model C centers to be far away from high population density areas. Space plays important role in the Model C—thus the model uses suburban areas or large parking lots in urban settings [11, 12]. To prevent contamination, HCWs should change personal protective equipment (PPE) after completing screening for each patient. In Model C, HCWs are able, on average, to obtain samples from 6 to 8 individuals per hour.
- *Model D* Operates with a “Glove-based wall system” that separates those being tested from HCWs conducting the tests. Those waiting for tests must wait outside the screening center, as in model B. By separating the screening areas from waiting areas, Model D centers minimize the risk of transmission. Outside the screening centers, organizers ask those waiting to maintain a minimum of 2 m from any other person to minimize the risk of transmission from droplets. Because HCWs collect samples wearing plastic gowns, N95 masks, and goggles, Model D does not require wearing of coveralls. Here HCWs test an average of 2–3 individuals per hour. South Korea designed these centers to accommodate those at high risk of COVID-19 transmission so they do not go other COVID-19 screening centers [13].
- *Model E* Operates at Incheon Airport COVID-19 to test pedestrians arriving on airplanes. Model E uses booths similar in appearance to the pay phone booths. Individuals to be tested enter the booths, and HCWs obtain the samples while standing outside the booths. Subsequently, a specially designated cleaning person (a different HCW) disinfects the empty booths while HCWs obtain samples from others [14]. HCWs visit multiple screening booths for sample collection. For model E, HCWs collect samples, on average, from 10 to 12 individuals per hour, more than for the other models (Table 1).

**Table 1:** The main features of COVID-19 screening centers in South Korea

	Model A	Model B	Model C	Model D	Model E
Location	Inside	Outside	Outside	Outside	Inside
Open time	09:00–18:00	day and night (24 h)	8:30–17:00	09:00–18:00	day and night (24 h)
Specimen collection (per hour)	1 individuals	2 individuals	6–8 individuals	2–3 individuals	10–12 individuals
HCW	–Model A, Model B: depends on the size of screening center, at least 2 HCW (doctor or nurse) –Model C: 4–8 HCW (1) Doctor: 1–2 (2) Nurse: 1–2 (3) Administrative worker: 1–3 (4) Quarantine(disinfection): 1 –Model D: at least 2 HCW in each booth (doctor or nurse) –Model E: at least 3 HCW (doctor or nurse)				
Necessary PPE	(1) KF94 respiratory mask or N95 mask (2) Goggles (3) Disposable body coveralls (4) Disposable gloves (5) Disposable shoes (6) Disposable plastic gowns (only Model D)				
Medical equipment	(1) X-ray machine (2) Negative pressure “Tents” (3) Testing kits (4) Glove-based wall (only Model D)				

*HCW* health care worker, *PPE* personal protective equipment

## Discussion

On World Malaria Day 2012, the World Health Organization (WHO) launched an initiative called ‘T3: Test, Treat, Track’ [15]. After 'testing', it is important to quarantine confirmed cases for initial management of infectious diseases. According to Yamey, South Korea flattened the curve through rapid and bold actions, exemplified by establishment of screening centers, including drive-through centers, that enabled large-scale testing [16]. South Korea focused on screening centers to prevent COVID-19. (see Fig. 2 for the distribution of these screening centers across regions of South Korea.) The government quickly installed general COVID-19 screening centers connected with public health centers, public hospitals, and private hospitals. This allowed for COVID-19 screening centers to be operated in all administrative districts. And the general centers systematically helped to prevent community spread across the country. Previous studies showed the necessity to prevent spread of COVID-19 in areas with high population density through control measures [17–19]. For this reason, South Korea appeared to distribute the general and DT screening centers in Seoul, Gyeonggi-do, and six large cities with high population density. The spread of COVID-19 is related to traffic volume and transportation trends [20]. Both traffic volume and population density appear to have influenced locations for the DT screening centers. The Namhae Highway, that connects Gyeongsangnam-do and Jeollabuk-do, carries a high volume of traffic [19]; thus these DT screening centers would enable efficient screening of people in automobiles. Distribution of DT screening centers in central South Korea along the Gyeongbu Highway addressed the route with the highest volume of traffic and enabled those in cars to be tested for COVID-19 at these sites. Only one DT screening center operated in Gangneung, and none in Gangwon-do. Gangwon-do is close to Seoul and Gyeonggi-do, and connected to the Yeongdong Highway that carries the second highest traffic volume in South Korea [21]. Establishment of more DT screening centers in the areas with high population density around the Yeongdong Highway would facilitate better geographic balance in distribution of COVID-19 screening centers. If resources for establishing screening centers are limited and the screening centers cannot be established in all the areas, a local screening cooperation system, as implemented by Goyang city in Gyeonggi-do could also be used to make the screening centers with different roles [22]. The Cooperation system worked with local clinic or health center and COVID-19 public relief hospital, connecting information about COVID-19 screening cases.

South Korea is improving COVID-19 screening center models to prevent secondary infections between HCWs conducting COVID-19 screening and those they test, and to enable rapid and efficient screening. Model A is not adequate to manage highly infectious diseases, such as COVID-19. South Korea implemented Model B in the early stage of COVID-19 transmission to manage this highly infectious disease. Screening centers located outside the hospital buildings or public health centers can be used for rapid responses to infectious diseases because those to be tested must wait outside these screening centers. Thus, risk of secondary infection is lower in model B than in model A. However, model B requires more time to collect each sample, and HCWs must change all PPEs (coveralls, plastic gowns, and N95 masks) after each test. This leads to high levels of fatigue in HCWs [23]. Because those waiting for tests line up without implementing ‘Social distancing through markings on the ground’, the risk of droplet infection is also very high [24, 25].

Model C, a now famous model of screening centers developed in South Korea, proved to be efficient, effective, and safe in many countries across Europe, the USA, and Latin America [26]. South Korea (Fig. 2) distributed model C screening centers in the outskirts of cities with high population density, and along the highways with high volumes of traffic. Model C screening centers serve well where traffic volume is high. Model C can be useful in countries with high population density, such as South Korea, and where a vast expanse of land may hinder effective management of screening centers, as in the USA, Australia, and China. Because cars serve as the isolation booths, implementation of model C can help to prevent secondary transmission among those waiting. HCWs should still change all the PPE after each screening to prevent contamination. Increased fatigue in HCWs remains as a limitation of this model.

Model D may be necessary at local healthcare facilities, such as public health centers and their branch offices. Although DT screening centers (Model C) ensure safe and convenient sample collection, they depend on those being tested arriving in their own cars. Therefore, those who do not own cars will visit other models of COVID-19 screening centers closest to where they live. Establishment of ‘Glove-walled WT COVID-19 screening centers’ at local healthcare facilities may be effective for safe management of those waiting for tests and prevent community-acquired infections [13, 27]. Because HCWs who screen these centers are also protected from the infection and experience less fatigue in the process, this model may be the most effective.

South Korea has not closed its borders for COVID-19 prevention. Instead, it tests all those entering from other countries and COVID-19 transmission through imported cases is rare in South Korea. Walk-through COVID-19 screening centers at Incheon Airport in South Korea prevent transmission of infectious diseases from those entering from other countries. The use of multiple COVID-19 testing booths and fast sample collection makes this possible.

Even if South Korea manages efficiently the confirmed COVID-19 cases, secondary transmission may still occur, most likely from imported cases. Therefore, model E is the most effective type of screening center for preventing secondary transmission of COVID-19 through importation. Its use will address problems associated with economic and social isolation resulting from closed borders.

## Conclusions

Efficient and effective management of COVID-19 screening centers and of the screening system and its use for mass COVID-19 testing was a major reason for South Korea’s success flattening the curve on COVID-19 cases.

Geographic distribution of COVID-19 screening centers shows concentration around big cities with high population density. Drive-through screening centers operate around big cities and along the major highways with high volumes of traffic. Some areas may require more drive-through screening centers, and better balance in distribution of COVID-19 screening centers across the country.

For infectious diseases with high risk of infection, such as COVID-19, advanced screening centers (models C, D, and E) proved to be more effective and efficient in the prevention of COVID-19 than the traditional screening centers (models A and B). Drive-through centers might be most effective in large countries or in the outskirts of cities. In addition, the ‘glove-walled walk-through screening centers’ can be effective at public health centers or their branches in local communities. Even without blocking borders, South Korea’s aggressive screening at Incheon Airport in South Korea showed outstanding results for preventing the transmission through importation and will be effective at airports or other ports.

South Korea benefited from establishing COVID-19 screening centers in all administrative areas to prevent the spread of community infection. It will be important elsewhere, as in South Korea, to establish an infectious disease delivery system that can lead to 'Test-Treat-Track'.

## Footnotes

1 Adapted from Kwon et al. [11].
